# Longitudinal Changes in Clock Drawing Test (CDT) Performance before and after Cognitive Decline

**DOI:** 10.1371/journal.pone.0097873

**Published:** 2014-05-29

**Authors:** Ping Wang, Langfeng Shi, Qianhua Zhao, Zhen Hong, Qihao Guo

**Affiliations:** Department of Neurology, Huashan Hospital, Shanghai Medical College, Fudan University, Shanghai, China; “Mario Negri” Institute for Pharmacological Research, Italy

## Abstract

**Background:**

Many scoring systems exist for clock drawing task variants. However, none of them are reliable in evaluating longitudinal changes of cognitive function. The purpose of this study is to create a simple yet optimal scoring procedure to evaluate cognitive decline using a clinic-based sample.

**Methods:**

Clock-drawings from 121 participants (76 individuals with no dementia and later did not develop dementia after a mean 41.2-month follow-up, 45 individuals with no dementia became demented after a mean 42.3-month follow-up) were analyzed using t-test to determine a new and simplified CDT scoring system. The new scoring method was then compared with other commonly used systems.

**Results:**

In the converters, there were only 7 items that are significantly different between the initial visits and the second visits. We propose a new scoring system that includes the seven critical items: numbers are equally spaced (12–3–6–9) (p = 0.031), the other eight numbers are marked (p = 0.022), numbers are clockwise (p = 0.002), all numbers are correct (p = 0.030), distance between numbers is constant (p = 0.016), clock has two hands (p = 0.000), arrows are drawn (p = 0.003). Compared with other traditionally used scoring methods, this based change clock drawing test (BCCDT) has one of the most balanced sensitivities/specificities with a clinic-based sample.

**Conclusions:**

The new CDT scoring system provides further evidence in support of a simple and reliable clock-drawing scoring system in follow-up studies to evaluate cognitive decline, which can be used in assessing the efficacy of medicine.

## Introduction

Neuropsychological evaluations are an integral part of a complete geriatric evaluation used to diagnose dementia. The clock drawing test (CDT), widely acknowledged for its simplicity and ease of administration, is a measure used to detect cognitive decline associated with a variety of neurobehavioral disorders. Moreover, the CDT requires different cognitive abilities including auditory and visual comprehension, concentration, visuospatial abilities, abstract conceptualization, and executive control [1]. Deficits in these areas reflect possible frontal and temporoparietal disturbances that are often exhibited in Alzheimer disease (AD) [2]–[4], and that may not easily be detected by commonly-used cognitive screening tests such as the Mini-Mental State Exam (MMSE) [5].Correlating highly with the MMSE [6] and other measures of global cognitive decline, the CDT serves as a simple and nonthreatening cognitive screen, rendering it a popular tool in both clinical and research practices [5],[7].

In the past 30 years, many variations of the Clock Drawing Test (CDT) have risen to the forefront as a dementia screening measure [6], [8]–[17] (see table 1). The scoring system by Shulman et al. in 1986 [18] was one of the oldest methods. Sunderland et al. [9] used a 10-point anchored system based on preset criteria with an arbitrary cut-off at 6 points. They found that interrater reliability was high in clinicians and non-clinicians. However, this scale proved difficult to apply according to the criteria provided since it assumes that the representation of the hands is first and entirely affected, and other errors in the representation of numbers and the clock face occur later. Therefore, some drawings received very low scores for minor errors in the representation of numbers even though the hands were properly placed. In 1989, Wolf-Klein et al. [6] tested patients who were admitted consecutively to a nursing facility without preselection, although the group with AD was older than the normal group. The 10 anchor points pertain only to the spacing of the numbers; time setting is not assessed, therefore their system was less sensitive to problems with executive functioning. Sample anchor points include: 10 ‘normal’; 7 ‘very inappropriate spacing’; 4 ‘counter- clockwise rotation’; and 1 ‘irrelevant figures’. They reported a sensitivity of 75.2% and a specificity of 97.7% for distinguishing between demented and “mentally normal elderly.” The Clock Completion Test of Watson et al. [11] is an objective and simple scoring method. The subject is asked to place all the numbers in the clock, but not to set a time. Consequently, the scoring is only based on the position of the numbers in the clock face. No hands are required or scored and so some sensitivity is lost. The authors report that the number of digits in the 4th quadrant (9–12) had the best agreement with the diagnosis of dementia. The Clock Drawing Interpretation Scale (CDIS) by Mendez et al. [10] uses 20 points distributed between general impression, placement of numbers and hands with a score of higher than 18 as being normal. The authors found that the presence of the number 2 and the correct location of the minute hand were the items most frequently absent and were absent in all AD patients. In 1998, Royall et al. [14] developed the CLOX test, a CDT scoring system, which they mention is specifically designed to measure executive control functions. The patient is asked to draw a clock on an empty page and later to copy a clock. The authors suggest that the difference between these tests can be a measure of executive control function.

**Table 1:** Characteristics of clock drawing test scoring systems.

Reference	Pre-drawn clock	Time setting	Scoring criteria and range	Correlation with other measures
Shulman et al. (1986)	Yes	11:10	5 points awarded for “perfect” clock, 4 points for clock containing minor visuospatial errors, 3 points for acceptable visuospatial organization but inaccurate representation of 10 past 11, 2 points for moderate visuospatial disorganization of numbers, 1 point for a severe level of visuospatial disorganization and 0 points for inability to make any reasonable attempt.	MMSE = −0.65, SPMSQ = −0.66, GDS = −0.32
Sunderland et al. (1989)	No	2:45	10-point scoring system with 1 as the lowest score and 10 as the highest score. 5 points given for accurate drawing a clock face with numbers placed correctly; remaining 6–10 points awarded for accuracy of hands denoting the time 2:45. Cut-off score of 6/10 indicates normal cognitive functioning.	GDS(r = 0.56),DRS(r = 0.59), BDRS(r = 0.51), SPMSQ(r = 0.59,p<0.001)
Wolf-Klein et al. (1989)	Yes	No	10-point system with scores corresponding to 10 hierarchical clock patterns from a previous pilot study. Cut-off score of less than 7 indicating “abnormal.”	Not assessed
Watson et al. (1993)	Yes	No	Clock is divided into four quadrants with the greatest weight assigned to the fourth quadrant (numbers 9–12). Each error falling into quadrants one, two and three contributes a score of 1, and each error in the fourth quadrant contributes a score of 4. Score of 0–3 indicates normality, whereas a score of 4 or greater indicates abnormality.	Not assessed
Mendez et al.(1992)	No	11:10	20-item scale with each clock attribute independently scored as a dichotomous variable. Attributes based on analysis of frequency of errors in clock drawing test.	Rey Complex Figure = 0.66, Symbol Digit = 0.65, MMSE = 0.45, GDS = 0.40
Royall et al.(1998)	No	1:45	Maximum score on the drawing task (CLOX 1) is 15 points. Maximum score on the copying task (CLOX 2) is 15 points. Lower scores indicate impairment. Cut-off scores of 10/15 (drawing task) and 12/15 (copying task) to indicate normal functioning. Points are awarded based on the answers to a set of 15 questions (e.g., Does figure resemble a clock? Outer circle present?)	EXIT25(r = 0.78, p<0.001), MMSE (r = 0.76, p<0.001)
Rouleau et al. (1992)	No	11:10	10-point scale that independently assesses three subscales: (1) representation of clock face (maximum of 2 points); (2) layout of numbers (maximum of 4 points); and (3) position of hands (maximum of 4 points). Lower scores indicate greater impairment.	Not assessed
Tuokko et al.(1992)	Yes	11:10	Errors on clock drawing categorized into the following classes: perseverations, omissions, rotations, misplacements, distortions, substitutions and additions. Greater than two errors on clock drawing considered abnormal. Clock setting and clock reading achieve a maximum of 3 points. Greater than 2 errors is considered a positive (abnormal) result for clock drawing, whereas the cut-off for the clock setting and clock reading tasks was a score of less than 13.	Not assessed
Manos and Wu (1994)	Yes	11:10	10-point system with a transparent circle divided into eighths that acts as a scoring tool for the drawn clock. Points are awarded based on the numbers falling into their proper section and accuracy of hands. Cut-off score of 7/10 used by authors to indicate a “normal” clock.	Trail Making Test Part A(r = 0.48, p<0.001), MMSE(r = 0.50, p<0.001), Block Design Test(r = 0.56, p<0.001)
Lessig et al. (2008)	No	8:20 or 11:10	Analyzed three existing scoring systems [8], [10], [13] to isolate six specific errors that were best able to discriminate patients with dementia from those without. A final algorithm was created from these six errors: inaccurate time setting, missing hands, missing numbers, number substitutions or repetitions, and failure to attempt clock drawing. If any error was identified, the clock was classified as abnormal.	Not assessed

Recently, more and more researches focused on its utility on screening mild cognitive impairment despite the inconsistent results, but little is known about the longitudinal changes in performance before and after cognitive decline. To our knowledge, most of the previous articles were cross-sectional, no article has evaluated whether individual with no dementia had progressed to mild Alzheimer's disease using CDT. Therefore, we conduct this study to investigate which aspects of clock drawing are important factors while assessing the characteristic changes in performance over time.

## Methods

### Participants

This study was conducted at the Memory Clinic of Shanghai Huashan Hospital Fudan University. The cohort consisted of participants referred to the clinic between June 2004 and Nov 2012 after they had finished the laboratory tests and cranial CT/MRI scan and were found to have no clinically significant abnormalities in vitamin B12, folic acid, thyroid function (free triiodothyronine-FT3, free tetraiodothyronine-FT4, thyroid stimulating hormone-TSH), rapid plasma regain (RPR), or treponema pallidum particle agglutination (TPPA). During the initial visits, all patients were assessed by physicians experienced in dementia disorders, and underwent thorough physical, psychiatric and neurological examinations, as well as an interview that focused on their cognitive symptoms. All of the MCI participants were diagnosed according to the following which take Mayo criteria [37] as reference: (1) cognitive complaints verified by an informant; (2) cognitive impairment lasting more than 3 months; (3) mini-mental state examination-Chinese version (C-MMSE) ≥ cut-off score for adjusted education: edu≥9 yr, 26; 6≤edu<9 yr, 22 [19]; (4) preserved basic ability of daily life (ADL)/minimal impairment in complex instrumental functions; (5) etiology unknown; (6) normal hearing and sight; (7) have not met the diagnostic criteria for dementia based on the criteria from the National Institute of Neurological and Communicative Disorders and Stroke and the Alzheimer's Disease and Related Disorders Association (NINCDS-ADRDA).

In the present study, 121 participants at baseline were included. The participants were followed about four years after the first visits. 76 participants did not convert to dementia over longitudinal follow-up with a mean duration of 41.2 months. These participants are termed Non-converters (mean age = 68.8 years, SD = 9.0; mean education = 13.4 years, SD = 2.6). Another group of 45 participants progressively deteriorated and were judged clinically as having developed Alzheimer' s Disease over longitudinal follow-up. They are termed Converters (mean age = 69.4 years, SD = 6.8; mean education = 12.6 years, SD = 2.3). The mean duration of follow-up for the converters was 42.3 months (SD = 18.8). AD was diagnosed as probable AD according to the National Institute of Neurological and Communicative Disorders and Stroke-Alzheimer's Disease and Related Disorders Association (NINCDS-ADRDA/NINCDS-AIREN) criteria. According to the scores he/she obtained in MMSE(Mini-Mental state examination) [20], CFT (complex figure test) [21], AVLT(auditory verbal learning test) [22], AFT(animal fluency test) [23], STT(shape trails test) [24], CDR(clinical dementia rating scale) [25] , SCWT(Stroop color word test) [26] at the both visits, the severity of AD was just mild. This study was approved by the ethics committee of Shanghai Huashan Hospital Fudan University. All participants signed a consent form.

### Procedure

To determine the general cognitive function, all study subjects completed MMSE(Mini-Mental state examination) [20], CFT (complex figure test) [21], AVLT(auditory verbal learning test) [22], AFT(animal fluency test) [23], STT(shape trails test) [24], CDR(clinical dementia rating scale) [25], SCWT(Stroop color word test) [26] at the both visits.

During the clock-drawing test, participants were asked to draw a big circle and put the numbers of the clock, and then they were asked to indicate the time as “50 after 13.” There was no time limit for this test.

According to previous studies, we chose 18 items and classified them into three major components: (a) drawing planning; (b) numbering; (c) placement and size of the hands. Each category can be further subdivided into some aspects. Within this study, we scored each clock according to the 18 items, by rating 1 if correct and 0 if wrong.

Moreover, five different scoring systems were used to score each clock blinded to the results of the rest of the assessment. We chose them because they were simple, representative and took the physicians less time. The three semi-quantitative scoring systems (Sunderland et al., 1989; Shulman et al., 1993; Watson et al., 1993) focused on scoring the whole clock, while the two quantitative scoring systems (MOCA-CDT, 2005 [38]; Pfizer Inc. and Eisai Inc) focused on different aspects of the clocks (such as clock face, numbers or hands) and scored them separately. The scoring methods used in this study are as follows: (1) The CDT by Sunderland et al.: 10 ‘hands are in correct position’; 7 ‘placement of hands is significantly off course’; 4 ‘further distortion of number sequence’; and 1 ‘either no attempt or an uninterpretable attempt is made’; (2) The CDT by Watson et al.: a clock is divided into quadrants and a score of 1 point is given for any error in the first 3 quadrants and 4 points for any error in the 4th quadrant; (3) The CDT by Shulman et al.: this scoring method has been better at predicting AD compared to other scoring methods [9]. Five points were given for a perfect clock and 0–4 points depending on the severity of the errors; (4) The CDT by Pfizer Inc. and Eisai Inc: clock scoring criteria are basic with 1 point per clock contour, numbers, numbers' location and hands; (5) MOCA-CDT: the 3-item scoring system scores range from 0 (normal clock) to 3 (severe impairment). Within the MoCA, clock drawing is one test item involving 3 of the total 30 points possible. Likely to maximize clinical time, clock scoring criteria are basic with 1 point per clock contour, numbers and hands.

### Data analysis

Initial analyses (t test) examined the relationship between cognitive status (non-converter vs. converter) and age, years of education or interval between the two assessments to determine if these variables should be considered as covariates.

MMSE total, CFT-Copy, CFT-Recall, AVLT-I, AVLT-II, AFT-total, STT-A, STT-B, CDR, CDR-SB were compared between non-converters at the first visit (V1) and non-converters at the second visit (V2), converters V1 and converters V2, as well as non-converters V2 and converters V2 to determine the general cognitive function.

We selected 18 items from CDT associated with dementia according to previous studies. Each of the 18 items was converted to a dichotomous variable (0, 1) with “0” indicating no and “1” indicating yes. In order to understand if any of the items could predict cognitive status (non-converter vs. converter), an initial t test was conducted between non-converters V1 and converters V1. To find the longitudinal changes in performance before and after cognitive decline, a second t test was then conducted between V1 and V2 in converters. Moreover, we conducted another t test between non-converters V2 and converters V2 to know the differences of the 18 items between the patients with dementia and the patients with no dementia.

Once the items that significantly discriminated between converters V1 and converters V2 had been isolated, we proposed a new scoring system named as based change clock drawing test (BCCDT). Then we compared the BCCDT with the CDT by Sunderland et al., the CDT by Watson et al., the CDT by Shulman et al., the CDT by Pfizer Inc. and Eisai Inc and MOCA-CDT.

For all of the 242 assessments, the CDT scores obtained from the six scoring methods were correlated with each other to investigate the relationship between the types of scoring method.

Comparison for continuous variables was evaluated with the Student t-test or the Mann-Whitney U test when the data were not normally distributed.

P values and CIs were estimated in a 2 –tailed fashion. Difference was considered to be statistically significant at P<0.05.

Data were analysed using statistical software (SPSS 13.0; Chicago, Illinois, USA).

## Results

### 1. Characteristics of the participants

During clinical follow-up, 76(63%) participants remained non-demented and 45(37%) participants developed dementia. We divided all of the participants into two groups, Non-converters and Converters. Initial T test revealed that age (t = 0.340, P = ns), education (t = 1.726, P = ns) or interval between two assessments (t = 0.349, P = ns) had no significant impact on the results (see table 2).

**Table 2:** Age, education, and interval between two assessments for individuals classified with Non-converters and Converters.

	Non-converters(n = 76)	Converters(n = 45)	t(P)
Age at baseline (year)	68.8 (9.0)	69.4 (6.8)	0.340(0.735)
Education(year)	13.4 (2.6)	12.6 (2.3)	1.726 (0.087)
Interval between two assessments (month)	41.2 (16.7)	42.3 (18.8)	0.349 (0.728)

### 2. Cognitive state of the Non-converters and Converters

According to the cognitive state at V2, we got four groups as follows: Non-converters V1, Non-converters V2, Converters V1 and Converters V2. The first two meant baseline visit and second visit of the participants who did not convert to dementia over longitudinal follow-up, and the latter two indicated first visit and second visit of the participants who developed Alzheimer' s Disease over longitudinal follow-up. We used T test to compare MMSE total, CFT-Copy, CFT-Recall, AVLT-I, AVLT-II, AFT-total, STT-A, STT-B, CDR, CDR-SB between two of the four groups. The result showed that most had significant difference, except for CFT-Copy between Non-converters V1 and Converters V1, STT-B and CDR between Converters V1 and Converters V2 (see table 3).

**Table 3:** Cognitive state of the four groups.

Index	Non-converters(n = 76)		Converters(n = 45)		Comparison t(P)		
	V1	V2	V1	V2	Non-converters V1 vs Converters V1	Converters V1 vs Converters V2	Non-converters V2 vs Converters V2
MMSE total	27.4 (1.7)	27.6 (2.1)	26.0 (1.5)	21.8 (4.1)	4.627 (0.000)	6.192 (0.000)	8.658 (0.000)
CFT-Copy	33.1 (4.5)	33.1 (2.7)	31.3 (4.9)	22.2 (11.7)	1.977 (0.051)	4.655 (0.000)	5.942 (0.000)
CFT-Recall	12.8 (8.3)	12.4 (7.9)	6.0 (6.4)	3.4 (4.8)	4.956 (0.000)	2.096 (0.039)	7.646 (0.000)
AVLT-I	14.4 (4.9)	17.0 (6.1)	11.4 (3.3)	8.6 (4.0)	4.023 (0.000)	3.439 (0.001)	8.955 (0.000)
AVLT-II	3.2 (3.1)	4.8 (3.4)	1.3 (1.7)	0.4 (0.9)	4.389 (0.000)	3.032 (0.003)	10.345 (0.000)
AFT-total	15.4 (4.4)	15.2 (4.7)	11.3 (3.6)	9.0 (4.6)	5.499 (0.000)	2.565 (0.012)	6.948 (0.000)
STT-A	59.8 (20.3)	68.3 (26.0)	72.2 (30.9)	126.7 (66.4)	2.375 (0.021)	4.842 (0.000)	5.470 (0.000)
STT-B	175.7 (83.0)	177.9 (62.9)	219.2 (103.0)	238.2 (61.5)(N = 34)	2.391 (0.019)	0.947 (0.347)	4.694 (0.000)
CDR	0.5(0.2)	0.5 (0.2)	1.0(0.3)	1.1 (0.6)	8.485 (0.000)	1.290 (0.202)	3.781 (0.001)
CDR-SB	2.5 (1.6)	2.9 (1.9)	3.9 (1.7)	6.0 (4.4)	4.047 (0.000)	2.129 (0.044)	3.160 (0.004)

P<0.05 is significant. MMSE total: total score of Mini-Mental state examination; CFT-Copy: copy part of complex figure test; CFT-Recall: recall part of complex figure test; AVLT-I: immediately recall of auditory verbal learning test; AVLT-II: delayed recall of auditory verbal learning test; AFT: animal fluency test; STT-A: part A of shape trails test; STT-B: part B of shape trails test; CDR: clinical dementia rating scale; CDR-SB: clinical dementia rating scale Sum of Boxes.

### 3. Significant and non-significant items

Table 4 shows the T test conducted to assess the utility of 18 items in our sample. Firstly, there was only one significant item at baseline between converters and non-converters (t = 4.731, p = 0.030), and performance in the converters were better than that in the non-converters, meaning that it was difficult to predict dementia. Secondly, for the items that were poorly finished, the accuracy rate of which was lower by 50% at baseline in converters, including “12, 3, 6, 9” are first written after the circle, “1, 2, 4, 5, 7, 8, 10, 11” are equally spaced, hour hand is towards correct number, minute hand is towards correct number, minute hand is longer than hour hand, there was no significantly difference between V1 and V2, showing that poorly finished items at baseline were not always the sensitive one to predict dementia. Thirdly, in the converters, there were four items, the score of which was higher in V2 than in V1, indicating that those four items were not helpful to improve forecast value. Therefore the total score of CDT should not just be the addition of each item. Fourthly, at the second visit, there were 15 items that were significantly different between non-converters and converters. But among the converters, there were only 7 items that could tell differences between V1 and V2, which means when comparing dementia with no dementia, the sensitive items between cross-sectional comparison and longitudinal comparison were not the same. Finally, there were seven significant items that appeared to be possible markers of progression to dementia in follow-up studies. Numbers are equally spaced (12–3–6–9) (p = 0.031), the other eight numbers are marked (p = 0.022), numbers are clockwise (p = 0.002), all numbers are correct (p = 0.030), distance between numbers is constant (p = 0.016), clock has two hands (p = 0.000), arrows are drawn (p = 0.003), all parameters indicated remarkable differences between baseline and follow-up scores in converters. The conclusion that can be drawn here is that these seven items may consist of a simple clock-drawing scoring system in follow-up studies to evaluate whether individual with no dementia had progressed to dementia. We named the new scoring system based change clock drawing test (BCCDT). Another 11 items no longer proved to be major contributors.

**Table 4:** Initial clock-drawing items used in statistical analyses.

items	Non-converters (n = 76)		Converters (n = 45)		Comparison t(P)		
	V1	V2	V1	V2	Non-converters V1 vs Converters V1	Converters V1 vs Converters V2	Non-converters V2 vs Converters V2
Drawing planning							
1. The closed circle is first drawn	56(73.7)	69(40.8)	30(66.7)	28(62.2)	0.145(0.703)	0.391(0.532)	13.502 (0.000)
2. Number 12” is written before other numbers	66(86.8)	70(92.1)	40(88.9)	36(80.0)	1.656(0.198)	2.977(0.084)	2.764(0.096)
3. “12,3,6,9” are first written after the circle	40(52.6)	49(65.5)	17(37.8)	18(40.0)	1.781(0.182)	0.017(0.897)	6.136(0.013)
4. The center is approximately in the middle	60(78.9)	65(85.5)	32(71.1)	26(57.8)	0.233(0.630)	2.424(0.119)	10.633(0.001)
Numbering							
5. Numbers are inside the circle	61(80.3)	58(76.3)	33(73.3)	33(73.3)	0.130(0.718)	0.041(0.840)	0.005(0.942)
6. Numbers equally spaced (12–3–6–9)	40(52.6)	52(68.4)	31(68.9)	22(48.9)	4.731(0.030)	4.642(0.031)	3.859(0.049)
7. Numbers equally spaced (1–2–4–5–7–8–10–11)	26(34.2)	35(46.1)	16(35.6)	9(20.0)	0.138(0.711)	3.015(0.082)	7.741(0.005)
8. Another eight numbers are marked	65(85.5)	73(96.1)	37(82.2)	29(64.4)	0.049(0.825)	5.222(0.022)	20.835(0.000)
9. Numbers are clockwise	71(93.4)	74(97.4)	42(93.3)	34(75.6)	2.319(0.128)	9.032(0.002)	13.532(0.000)
10. Numbers have correct sequence	63(82.9)	71(93.4)	38(84.4)	33(73.3)	0.957(0.328)	2.912(0.088)	8.396(0.004)
*11. All numbers are correct	65(85.5)	71(93.4)	38(84.4)	31(68.9)	0.187(0.665)	4.099(0.030)	13.845(0.000)
12. Distance between numbers is constant	41(53.9)	48(63.2)	25(55.6)	14(31.1)	0.123(0.726)	5.791(0.016)	10.706(0.001)
Placement and size of the hands							
13. Clock has two hands	74(97.4)	70(92.1)	39(86.7)	23(51.1)	2.752(0.097)	16.685(0.000)	25.986(0.000)
14. Hour hand is towards correct number	45(59.2)	43(56.6)	18(40.0)	12(26.7)	3.184(0.074)	2.079(0.149)	9.510(0.002)
15. Minute hand is towards correct number	41(53.9)	55(72.4)	18(40.0)	21(46.7)	1.502(0.220)	0.306(0.580)	7.154(0.007)
16. Minute hand is longer than hour hand	41(53.9)	49(64.5)	19(42.2)	17(37.8)	0.958(0.328)	0.283(0.594)	7.380(0.007)
17. Arrows are drawn	50(65.8)	48(63.2)	27(60.0)	14(31.1)	0.068(0.795)	8.566(0.003)	10.835(0.001)
18. no additional hands are present	64(84.2)	63(82.9)	37(82.2)	37(82.2)	0.062(0.804)	0.099(0.753)	0.607(0.436)

If the answer is yes →1 point, if no →0 point;

*All numbers are correct: wrong numbers included cases of number that are missing, repeated, not include in 12-number range, or been substituted with some symbols.

### 4. Clock performance in relation to performance on other cognitive measures

Table 5 presents the correlation coefficients between the CDT score and other cognitive measures using correlation analysis. All correlations between the six CDT scores and the MMSE total score, AVLT-I, AFT and STT-A were significant, with the highest correlation occurring between BCCDT and MMSE total score, AVLT-I, AFT and STT-A. Sunderland scoring system and the BCCDT correlated with the time of Rey –O CFT-Copy (s), AVLT-II and correct number of SCWT-C, the highest correlation between Sunderland scoring system, BCCDT and time of Rey –O CFT-Copy (s), AVLT-II was obtained using BCCDT. Watson, Sunderland scoring system and BCCDT correlated with the CFT-Recall, and the three correlation coefficients were similar. MOCA-CDT, Shulman scoring system, Sunderland scoring system, and BCCDT correlated with time of SCWT-C(S), and the highest correlation was between BCCDT and time of SCWT-C(S). In conclusion, for BCCDT, it has displayed good correlation with other memory clinic measures (see table 5).

**Table 5:** Correlations between the CDT score and other cognitive measures.

	MMSE total	Rey –O CFT-Copy	Time of Rey –O CFT-Copy (s)	CFT-Recall	AVLT-I	AVLT-II	Time of SCWT-C(S)	Correct number SCWT-C	AFT	STT-A
MOCA-CDT	0.432**	0.626*	−0.096	0.098	0.306**	0.153	−0.268*	0.165	0.370**	−0.551**
Pfizer Inc. and Eisai Inc	0.406**	0.569**	−0.211	0.150	0.284**	0.199	−0.217	0.123	0.401**	−0.522**
Shulman	0.427**	0.634**	−0.138	0.190	0.237*	0.195	−0.244*	0.190	0.346**	−0.490**
Watson	−0.517**	−0.702**	0.206	−0.223*	−0.220*	−0.163	0.206	−0.071	−0.377**	0.622**
Sunderland	0.539**	0.644**	−0.269*	0.242*	0.389**	0.238*	−0.388**	0.279*	0.379**	−0.613**
BCCDT	0.551**	0.629**	−0.293*	0.222*	0.442**	0.250*	−0.402**	0.228*	0.465**	−0.693**

*Correlation is significant at the 0.05 level (two-tailed).

**Correlation is significant at the 0.01 level (two-tailed).

MMSE: Mini-Mental state examination; Rey –O CFT-Copy: copy part of Rey –O complex figure test; CFT-Recall: copy part of complex figure test; AVLT-I: immediately recall of auditory verbal learning test; AVLT-II: delayed recall of auditory verbal learning test; SCWT-C: part C of Stroop color word test; AFT: animal fluency test; STT-A: part A of shape trails test; BCCDT: based change clock drawing test.

### 5. Correlations between the six scoring methods

Table 6 summarizes correlations between the six scoring methods, including BCCDT. For the total 242 assessments, the six systems are moderately-to-highly correlated, with the highest correlation occurring between the MOCA-CDT and Pfizer Inc. and Eisai Inc scoring method. All correlations between BCCDT and others were statistically significant at the 0.01 level.

**Table 6:** Correlation coefficients between the six scoring methods based on the 242 assessments.

	MOCA-CDT	Pfizer Inc. and Eisai Inc	Shulman	Watson	Sunderland	BCCDT
MOCA-CDT	1	.907**	.877**	−.627**	.825*	.719**
Pfizer Inc. and Eisai Inc		1	.888**	−.692**	.820**	.784**
Shulman			1	−.697**	.896**	.781**
Watson				1	−.751**	−.748**
Sunderland					1	.788**
BCCDT						1

*Correlation is significant at the 0.05 level (two-tailed).

**Correlation is significant at the 0.01 level (two-tailed).

### 6. The utility of BCCDT comparing with other five scoring systems

T test was conducted to assess the utility of the six scoring systems. We found in converters, the scores at V1 and V2 was significantly different, and p value of BCCDT (p = 0.000) was the smallest in the six (see Table 7).

**Table 7:** Scores of four groups using six scoring systems.

	Non-converters (n = 76)		Converters (n = 45)		Comparison t(P)		
	V1	V2	V1	V2	Non-converters V1 vs Converters V1	Converters V1 vs Converters V2	Non-converters V2 vs Converters V2
MOCA-CDT	2.4 (0.6)	2.4 (0.6)	2.2 (0.5)	1.8 (0.9)	−1.517 (0.132)	2.435 (0.018)	4.014 (0.000)
Pfizer Inc. and Eisai Inc	3.0 (0.9)	3.2 (0.8)	2.8 (0.8)	2.4 (1.3)	−1.138 (0.258)	2.002 (0.049)	3.640 (0.001)
Shulman	3.8 (1.0)	3.9 (0.9)	3.3 (0.8)	2.7 (1.5)	−2.612 (0.010)	2.307 (0.024)	4.377 (0.000)
Watson	0.8 (1.9)	0.4 (1.2)	0.9 (1.5)	2.2 (3.0)	.224 (0.823)	−2.526 (0.014)	−3.713 (0.001)
Sunderland	8.5 (1.4)	8.7 (1.2)	7.9 (1.4)	6.0 (3.1)	−2.221 (0.028)	3.527 (0.001)	5.317 (0.000)
BCCDT	5.4 (1.4)	5.8 (1.3)	5.6 (1.4)	3.9 (2.4)	.940 (0.349)	3.989 (0.000)	4.635 (0.000)

### 7. Discrimination of different scoring systems between non-converters and converters

The area under the ROC curve is perhaps a more unbiased method to determine the efficiency of a screening test as it shows the relationship between sensitivity and specificity. ROC curves were drawn for the six scoring systems to evaluate their respective areas under the curve, sensitivities, and specificities (see Table 8). Using the optimal cut-off score of 5, the differences between the two groups were most discernible under BCCDT, according to the ROC curve (area under the curve = 0.713, p = 0.001), while the sensitivity and specificity remained at 78.6% and 57.1%, respectively. The Watson scoring method had the smallest area under the curve (0.571, p = 0.260). The MOCA-CDT and Shulman scoring systems had the highest sensitivities at 92.9% and 88.1%, respectively. BCCDT and Sunderland scoring procedures fell in the middle at 78.6%, and 73.8%, respectively. The Watson method had the lowest sensitivity at 54.8%, performing just above chance level for correctly identifying individuals with dementia. With regard to specificity, BCCDT scoring procedure had the highest specificity at 57.1%, followed by the Sunderland scoring method at 47.6%. The Pfizer Inc. and Eisai Inc's specificity closely trailed the Watson scoring system at 38.1%. Both the Shulman and MOCA-CDT procedures had the lowest specificities at 33.3% and 28.6%, respectively.

**Table 8:** Discrimination of different scoring systems between non-converters and converters.

	Area Under the Curve	Asymptotic Sig	Asymptotic 95% Confidence Interval	Cut-off	Sensitivity	Specificity
MOCA-CDT	0.601	0.110	0.479∼0.723	≤2	92.9	28.6
Pfizer Inc. and Eisai Inc	0.579	0.210	0.457∼0.702	≤3	69.0	38.1
Shulman	0.589	0.161	0.465∼0.713	≤3	88.1	33.3
Watson	0.571	0.260	0.447∼0.696	≥1	54.8	40.5
Sunderland	0.622	0.054	0.498∼0.746	≤8	73.8	47.6
BCCDT	0.713	0.001	0.602∼0.825	≤5	78.6	57.1

## Discussion

Using the clinical sample, BCCDT was found to be effective in evaluating the longitudinal changes in clock drawing test (CDT) performance before and after cognitive decline. It includes seven critical items (numbers are equally spaced (12–3–6–9), the other eight numbers are marked, numbers are clockwise, all numbers are correct, distance between numbers is constant, clock has two hands, arrows are drawn). Further investigations should examine these seven items in the context of other indicators of dementia such as story recall and the MMSE score.

MMSE is one of the most influential cognitive screening methods. It has been widely used in screening dementia and MCI. In previous studies, the orientation and delayed recall parts of the MMSE are good at predicting specifically AD [27], [28]. But with the clinical practice of MMSE, researchers found it was not sensitive enough to be used in follow-up of cognitive function. Recently, more and more researches focused on CDT, as it could reflect different cognitive abilities including auditory and visual comprehension, concentration, visuospatial abilities, abstract conceptualization, and executive control [1]. However, most were cross-sectional studies, there were few longitudinal studies. Ji et al. [29] described the longitudinal changes in performance and error types on CDT by dementia severity and subtypes. They concluded that longitudinal analysis of error on CDT may reflect different characteristics of cognitive deterioration according to dementia subtypes and dementia stages. Zhou [30] used Death scoring systems (total score of 4) to assess the efficacy of medicine, but the sensitivity and specialty has not been verified. Therefore, we hope a suitable CDT scoring system will help to evaluate cognitive function longitudinally. Lennie et al. [31] found that “the clock has two hands, the size difference of the hands is respected, and the hour hand is towards correct number” were three interesting findings that were early discriminators for developing dementia. These items may be good indicators of further cognitive decline. Sebastian et al. [32] concluded that the MMSE and the clock drawing test were as accurate as CSF biomarkers in predicting future development of AD in patients with MCI. But in our study, table 4 illustrated that there was only one significant item at baseline between the non-converters and converters, and performance in the converters were better than that in the non-converters, which means that it was difficult to predict dementia using any one of the 18 items.

Compared with the MCI-NP (participants with mild cognitive impairment who did not develop dementia on follow-up visits) group, participants in the MCI-D (participants with mild cognitive impairment who became demented after a 48-month follow-up) were more likely to fail the item for “size difference of the hands is respected.” [31] However, our results showed that poorly finished items at baseline were not the sensitive one to predict dementia.

A majority of studies focused on the utility of different CDT scoring system in screening dementia or MCI [33]–[35]. In this study, we found that when comparing dementia with no dementia, the sensitive items between cross-sectional comparison and longitudinal comparison were not the same. Therefore, the existing CDT scoring systems were not suitable for follow-up studies, and could not be used in assessing the efficacy of medicine.

According to the scores he/she obtained in MMSE (Mini-Mental state examination), CFT (complex figure test), AVLT (auditory verbal learning test), AFT (animal fluency test), STT (shape trails test), CDR (clinical dementia rating scale), SCWT (Stroop color word test) at both visits, the severity of AD was just mild. Patients who have been moderate to severe demented were not able to complete all of the tests. Therefore, BCCDT could be used to earlier recognize whether patients with MCI had progressed to mild AD. In addition, we discovered that the total score of CDT should not just be the addition of each item, as several items were not helpful to improve forecast value.

In comparing the non-converters and converters, the new scoring method with a cut-off score of 5 produced a sensitivity of 78.6% and a specificity of 57.1%. Even though the sensitivity of MOCA-CDT and the Shulman scoring method surpassed the new scoring system's sensitivity, the specificity of the new method was the highest among the six systems. This comparison revealed the new method to be more balanced than others for screening AD.

The correlation of the CDT with other screening tests, including the ‘gold standard’ MMSE, was good in most studies [15], [36], as well as in our study. We suggest that there may be a rationale for using both the MMSE and the CDT whilst evaluating longitudinal changes of cognitive function, as the MMSE measures are mostly verbal skills and so could not be sensitive enough. However, this would considerably increase the time of administration.

Because this was a longitudinal study, we may think that the performance decline in the seven items of BCCDT was due to aging. But in the non-converters, there was no significant difference between V1 and V2. Therefore, our results should not be interpreted as determining the effect of aging on CDT performance.

Several limitations of this study need to be considered when examining the results. The sample used in this study was not population based, but comprised clinic-based participants, which was not as ethnically diverse nor representative as might be desired. Results of the utility of our proposed scale should be verified in other population context to avoid the bias of “pre-selected patients”. There is no correlation analysis between the moment of making V2 and the moment of the diagnosis of dementia. Moreover, AD was diagnosed as probable AD according to the NINCDS-ADRDA/NINCDS-AIREN criteria, and there were no distinctive biomarkers such as beta-amyloid or position-emission tomography (PET), so error could not be avoided.

### Key points

Seven items of clock drawing test may consist of a simple clock-drawing scoring system in follow-up studies to evaluate whether individuals with no dementia had progressed to dementia.

It was difficult to predict dementia using the 18 items of clock drawing test.

Poorly finished items of clock drawing test were not always sensitive to predict dementia.

Some items of clock drawing test were not helpful to improve forecast value of dementia.
